# Application of novel nanocomposite-modified electrodes for identifying rice wines of different brands†

**DOI:** 10.1039/c8ra00164b

**Published:** 2018-04-10

**Authors:** Zhenbo Wei, Yanan Yang, Luyi Zhu, Weilin Zhang, Jun Wang

**Affiliations:** Department of Biosystems Engineering, Zhejiang University 866 Yuhangtang Road Hangzhou 310058 PR China jwang@zju.edu.cn

## Abstract

In this paper, poly(acid chrome blue K) (PACBK)/AuNP/glassy carbon electrode (GCE), polysulfanilic acid (PABSA)/AuNP/GCE and polyglutamic acid (PGA)/CuNP/GCE were self-fabricated for the identification of rice wines of different brands. The physical and chemical characterization of the modified electrodes were obtained using scanning electron microscopy and cyclic voltammetry, respectively. The rice wine samples were detected by the modified electrodes based on multi-frequency large amplitude pulse voltammetry. Chronoamperometry was applied to record the response values, and the feature data correlating with wine brands were extracted from the original responses using the ‘area method’. Principal component analysis, locality preserving projections and linear discriminant analysis were applied for the classification of different wines, and all three methods presented similarly good results. Extreme learning machine (ELM), the library for support vector machines (LIB-SVM) and the backpropagation neural network (BPNN) were applied for predicting wine brands, and BPNN worked best for prediction based on the testing dataset (*R*^2^ = 0.9737 and MSE = 0.2673). The fabricated modified electrodes can therefore be applied to identify rice wines of different brands with pattern recognition methods, and the application also showed potential for the detection aspects of food quality analysis.

## Abstract 2

In this paper, poly(acid chrome blue K) (PACBK)/AuNP/glassy carbon electrode (GCE), polysulfanilic acid (PABSA)/AuNP/GCE and polyglutamic acid (PGA)/CuNP/GCE were self-fabricated for the identification of rice wines of different brands.

## Graphical abstract

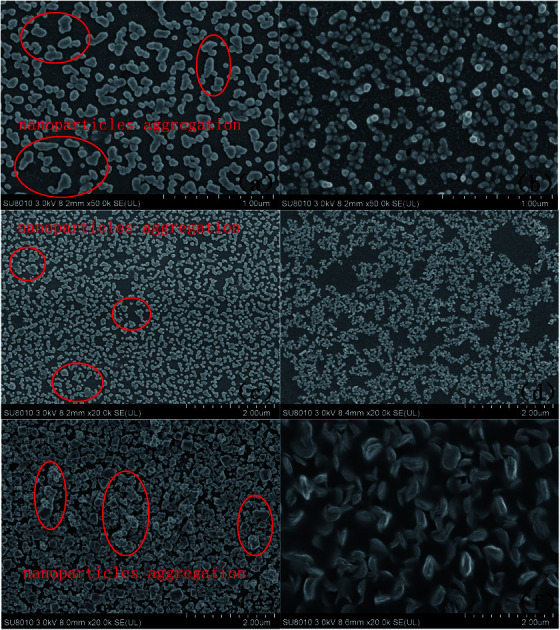

## 1. Introduction

An ideal analytical tool should be rugged, simple and reasonably affordable when applied to detect samples. In this regard, electrochemical sensors can offer the straightforward advantage of being able to distinguish one specific species in complex mixtures.^1–3^ In previous studies, glassy carbon electrode-modified chemically active materials showed high sensitivity to organic substances.^4–6^ Metal nanoparticle materials have attracted much research interest because of their good electrical conductivity and excellent catalytic properties, and electrochemical sensor-modified metal nanoparticles worked well in the determination of DNA, proteins, neurotransmitters, phytohormones and organic pollutants.^7–10^ However, these metal nanoparticles are unstable due to their high activity, easy oxidation and easy agglomeration due to van der Waals interactions.

Recently, numerous studies have focused on the synthesis of composite materials combining a polymer skeleton with incorporated inorganic structures.^11–13^ Polymers with conjugated chains are frequently used because of their low oxidation potential, electronic and ionic conductivity and porous morphology.^14,15^ Due to these advantages, a scheme was proposed using polymers as carriers to deposit metal nanoparticles in order to fabricate polymer/metal nanocomposite-modified electrodes. These modified electrodes can enhance the stability of the combination of the nanometre-sized material and electrode base, lessen the tendency for the particles to reunify and improve the catalytic properties of the nanocomposites.^16,17^

Wine brand is one of the most important attributes which highly influences wine selection,^18–20^ and many powerful brands such as Moët & Chandon have played an important role in building the reputation of wines. Wine dealers understand that this reputation building tends to originate from the special quality and taste of the wines, and brands can be identified as an indicator of the quality, reliability, security and safety of the product.

Chinese rice wine is a sweet, golden wine made from glutinous rice and wheat with a unique craft, and it is also one of the three most ancient alcoholic beverages in the world.^21–23^ During the last 10 years, a common fraudulent practice in the commercialization of Chinese rice wine is the sale of fake brands as real wine. The fake products, with no quality assurance, are dishonest and unfair for customers. Moreover, they can disrupt the normal market because of their lower price, and also cause customers to lose faith in the quality and taste of real wine. Traditional methods for the identification of wine brands mainly include sensory evaluation, physicochemical detection and precision instrument analysis.^24–26^ However, all of these methods have some limitations. Sensory evaluation is easily influenced by subjective and environmental factors, physicochemical detection is complicated and has high technical requirements, and the use of precision instruments requires complex pre-processing and has a high cost. Therefore, a simple, convenient and fast method is urgently needed for the identification of wine brands.

In past research, the applications of polymer/metal nanocomposite-modified electrodes focused on analyzing trace chemicals in complex mixtures.^27,28^ However, research into the use of nanocomposite-modified electrodes for identifying wines of different brands was rare. Through analyzing the characteristics and applications of polymer/metal nanocomposite-modified electrodes, they can be applied as a potential tool for identifying wine brands.

The previous research indicated that the three chemical substances, ascorbic acid (Asc, sour), tyrosine (Tyr, astringent) and gallic acid (GA, astringent), have good correlation with wine brands.^29,30^ In this study, three types of polymer/metal nanocomposite-modified electrode (PACBK/AuNP/GCE, PABSA/AuNP/GCE and PGA/CuNP/GCE), which have high sensitivity to Asc, Tyr and GA, respectively, were fabricated for the first time to identify rice wine brands. The characteristics of the modified materials were investigated using scanning electron microscopy (SEM). The working parameters of the modified electrodes were optimized by cyclic voltammetry (CV) and differential pulse voltammetry (DPV). Two types of multi-frequency large pulse voltammetry (MLPV), multi-frequency rectangle pulse voltammetry (MRPV) and multi-frequency staircase pulse voltammetry (MSPV), were applied to obtain the wine brand information using the chronoamperometric method. MRPV and MSPV were comprised of many frequency segments of rectangle and staircase pulses, respectively, and the principle of each frequency segment of MRPV and MSPV was the same as that of large pulse voltammetry. When a potential was stepped to the final potential from the base potential (normally 0 V), a sharp oxidized or reduced transient current from the Helmholz double layer next to the electrode surface would flow to the working electrode, until only the limited diffusion faradic current remained. The complete oxidation and reduction of the electroactive compounds next to the electrode surface needs a wide potential, and one shape of the pulse cannot meet this requirement. Using two types of pulse shape can provide a wider potential and more information on the oxidation (or reduction) of the electroactive compounds in the liquid samples. With the help of pattern recognition methods, the aims of this study were (1) to evaluate the performance of the three self-fabricated polymer/metal nanocomposite-modified electrodes for analyzing Asc, Tyr and GA, and (2) to identify rice wines of different brands using the three types of modified electrode based on classification and prediction methods.

## 2. Experimental

### 2.1. Reagents and instrumentation

ASC, GA, Tyr, acid chrome blue K (ACBK), sulfanilic acid (ABSA) and glutamate (GA) were purchased from Aladdin Chemical Co. Ltd., (Shanghai, China). HAuCl_4_ and CuSO_4_ were obtained from Sinopharm Chemical Reagent Co. Ltd., China. Phosphate buffer solution (PBS, 0.1 M) was prepared by mixing stock solutions of 0.2 M Na_2_HPO_4_·12H_2_O and NaH_2_PO_4_·2H_2_O. All reagents were of analytical grade and were used directly without purification, and all solutions were prepared with double-distilled water.

A pH meter (FE28-FiveEasy Plus, METTLER TOLEDO), electronic analytical balance (BS224S, Sartorius), ultrasonic cleaner (SK1200H, KUDOS), scanning electron microscope (SU8010, HITACHI, Japan) and magnetic stirrer (DF-101Z) were used. The fabrication of modified electrodes and rice wine identified experiments were performed on a PARSTAT 3000 A electrochemical workstation (AMETEK. Inc, USA). A conventional three-electrode system consisted of a working modified electrode, a platinum wire auxiliary electrode and a saturated Ag/AgCl reference electrode. A saturated KCl solution was used as the reference solution.

### 2.2. Rice wine samples

Rice wines of different brands, which include three types of Guyue Longshan (Dida (G-DD), Gold Five (G-GF) and Kucang (G-KC)), three types of Tapai (Techun (T-TC), Shougong (T-SG) and Laojiu (T-LJ)) and one type of Kuaijishan (K-HD), were all produced in Shaoxing (a city of Zhejiang province (120° 58′ E, 30° 01′ N), China). These rice wine samples were acquired from the manufacturers, and were kept in a cool and dry place (25 ± 1 °C) before analysis.

### 2.3. Preparation of the modified electrodes

In the study, three types of polymer/metal nanocomposite-modified electrode (PACBK/AuNP/GCE, PABSA/AuNP/GCE and PGA/CuNP/GCE), which have high sensitivity to ASC, GA, and Tyr, respectively, were fabricated for the first time.

Prior to modification, the GCEs were polished with 0.3 and 0.05 μm pasty slurry on chamois leather until a mirror-like surface was obtained, then the electrodes were rinsed gradually with absolute ethyl alcohol and deionized water for 2 min each. Afterwards, the pretreated GCEs were dipped into 0.5 M H_2_SO_4_ and scanned by repeated CV (at a scan rate of 100 mV s^−1^) ranging from −0.5 to 1.2 V until a steady state was established. Finally, the electrode surfaces were thoroughly washed with deionized water and dried in air.

For the fabrication of PACBK/AuNP/GCE, gold nanoparticles were electrochemically deposited onto the GCE surface in 1.2 mM HAuCl_4_ solution containing 0.1 M KNO_3_ for 240 s at a fixed potential of −0.2 V. The acquired Au/GCE was then electrodeposited in 0.1 M PBS (pH 6.0) containing 0.5 mM acid chrome blue K with a cycling potential from −1.6 to +2 V at a scan rate of 100 mV s^−1^ for 26 cycles.

For the fabrication of PABSA/AuNP/GCE, Au/GCE was made based on the steps described above. The Au/GCE was then electrodeposited in 2 mM sulfanilic acid containing 0.1 M PBS (pH 7.0) with a cycling potential from −1.5 to +2.5 V at a scan rate of 100 mV s^−1^ for 12 cycles.

For the fabrication of PGA/CuNP/GCE, gold nanoparticles were electrochemically deposited onto the GCE surface in 0.01 mM CuSO_4_ solution containing 0.1 M Na_2_SO_4_ with a cycling potential from −1.5 to 0 V at a scan rate of 20 mV s^−1^ for 10 cycles. The acquired Cu/GCE was then electrodeposited in 0.01 M glutamic acid solution containing 0.1 M PBS (pH 7.0) with a cycling potential from −0.8 to +2 V at a scan rate of 100 mV s^−1^ for 12 cycles.

### 2.4. Characterization of the modified electrodes and experimental procedure for the identification of wine brands

The general morphology of the modified electrodes was observed using a field-emission scanning electron microscope (SEM, SU8010, HITACHI). The electrochemical characterization of the modified electrodes in their various states (PACBK/AuNP/GCE, PABSA/AuNP/GCE and PGA/CuNP/GCE) was carried out using a traditional three-electrode system with CV. The modified electrodes acted as the working electrode, and a platinum counter electrode (purity 99.95%, length 10 mm, diameter 1 mm) and Ag/AgCl reference electrode (3.5 M saturated KCl, diameter 2 mm) were also included in the system.

During the experiment, a 40 ml sample of each type of rice wine was analyzed, and 0.1 M PBS (pH 2.0) was added in order to adjust the pH of the rice wine for the best detection conditions. The sample mixtures were analyzed by the modified electrodes with multi-frequency rectangle pulse voltammetry (MRPV) and multi-frequency staircase pulse voltammetry (MSPV). Both of the two potential waveforms, which were composed of three frequencies of 1 Hz, 10 Hz and 100 Hz, were increased from 0 V to the final potential (1.5 V) in steps of 0.25 V. For each sample, 3 replicates were prepared for analysis, and the average values of the replicates were used for the data evaluation. All experiments mentioned in this paper were performed at room temperature (25 ± 1 °C).

### 2.5. Pattern recognition methods

In the study, principal component analysis (PCA), locality preserving projection (LPP) and linear discriminant analysis (LDA) were applied for classification.^31–33^ Extreme learning machine (ELM), the library for support vector machines (LIB-SVM) and the backpropagation neural network (BPNN) were applied for prediction.^34–36^

PCA explains the maximum variance of the data set with the least number of major components without a significant loss of information.^37^ Supported by multivariate data analysis software, the samples receive new coordinates (scores) according to principal components describing the resumed sensor information.

LPP is a linear operation, and similarly to PCA, it can be very easy to determine the projection space of LPP by solving a generalized eigenvalue. As a result, the new samples can be directly projected onto this space, and it can maintain the maximum local characteristics of the original data.^38^

LDA is used as a supervised learning technique to extract features which preserve class separability. It provides a classification model, characterized by a linear dependence of the classification scores with respect to the descriptors.^39^

ELM was proposed by Huang *et al.*^40^ for reducing the time required to train single-layer feed-forward neural networks. It is only needed to set the number of hidden layer neurons without adjustment over the course of training to get the only optimal solution.^41^

LIB-SVM, which was proposed by Lin,^42^ is a library for support vector machines (SVMs). It is an optimized version based on standard SVMs and it is powerful for solving problems in nonlinear classifications, function estimation and density estimation.

BPNN is a multi-layer feed-forward network trained by the error backpropagation algorithm. It can learn and store a lot of input–output model mapping without revealing the mathematical equations of the mapping relationship in advance. BPNN has a strong nonlinear mapping ability and is especially suitable for classification or approximation.^43^

PCA, LPP and LDA were performed using SAS v8 (SAS Institute, Cary, NC, USA). ELM, LIB-SVM and BPNN were performed using Matlab 6.0. (MathWorks, Inc., USA).

## 3. Results and discussion

### 3.1. Characterization of the modified electrodes

**Fig. 1:**
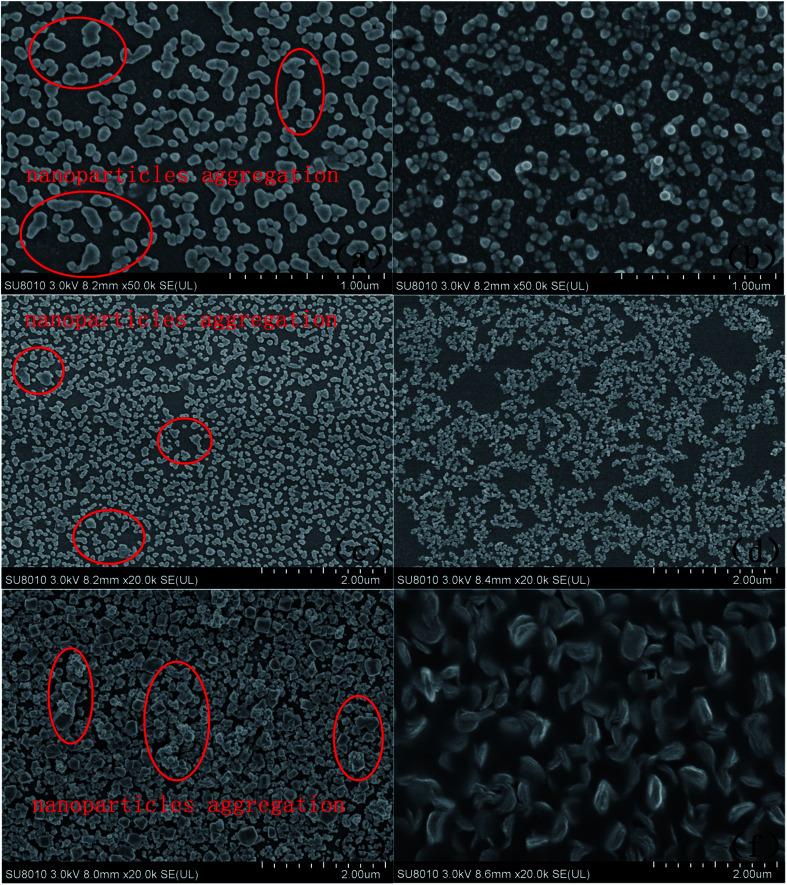
SEM images of AuNP/GCE (a), PACBK/AuNP/GCE (b), AuNP/GCE (c), PABSA/AuNP/GCE (d), CuNP/GCE (e) and PGA/CuNP/GCE (f).

SEM was used to observe the microstructure of the modified electrode surface. Fig. 1(a) and (b) show the typical SEM images of AuNP/GCE and PACBK/AuNP/GCE, respectively. As shown in Fig. 1(a), gold nanoparticles were clearly deposited on the GCE surface, but the nanoparticles varied greatly in size and obvious agglomeration had occurred. It can be seen from Fig. 1(b) that the PACBK film aggregated successfully on the surface of AuNP/GCE. Due to its catalytic properties, PACBK made the sizes of the gold nanoparticles homogeneous, and the agglomeration almost disappeared. Fig. 1(c) and (d) show the typical SEM images of AuNP/GCE and PABSA/AuNP/GCE. Although the AuNPs had deposited on the GCE surface successfully, the diameter of the agglomerated particles was up to 200 mm, which exceeded the general cognitional range of 1 to 100 nm for the AuNP diameter (Fig. 1(c)). Meanwhile, some agglomeration also occurred. After modification, it was obvious that the PABSA film had coated the electrode surface successfully. Moreover, the deposition optimized the configuration of the AuNPs, and there was no agglomeration of the AuNPs on the electrode surface (Fig. 1(c)). Fig. 1(e) and (f) show the typical SEM images of CuNP/GCE and PGA/CuNP/GCE. PGA had coated the surface of CuNP/GCE successfully. It not only performed its catalytic role but also improved the physical morphology of the CuNPs, changing their shape from grainy to willow leaf-shaped. The CuNPs with their new shape were more easily assembled on the surface of the electrode.

### 3.2. Electrochemical behavior of chemical substances at the modified electrodes

**Fig. 2:**
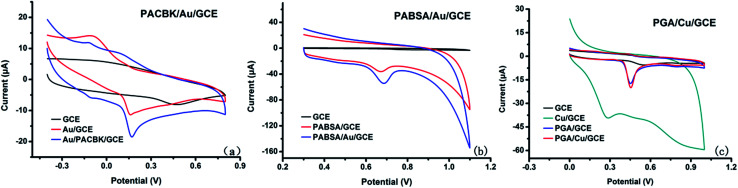
Cyclic voltammetry responses of (a) Asc sensors, (b) Tyr sensors and (c) GA sensors, modified with different materials.

The electrochemical behavior of Asc, Try and GA at the corresponding GCEs was investigated by cyclic voltammetry (CV). Fig. 2(a) shows the CV responses of Asc (0.32 mM) on different electrodes. The bare GCE presented no chemical response to Asc based on CV scanning, but there were electrochemical oxidation reactions on AuNP/GCE and PACBK/AuNP/GCE. Considering that the peak currents on the PACBK/AuNP/GCE surface were significantly larger than those on the AuNP/GCE surface, PACBK/AuNP/GCE presented better catalytic properties towards Asc.

Fig. 2(b) shows the CV responses of Tyr (0.1 mM) on different electrodes. GCE has a weak catalytic effect on Tyr, and there were no response currents on the GCE surface. After modification, the responses of PABSA/GCE and PABSA/AuNP/GCE showed an obvious increase. By comparing the oxidation peak currents of PABSA/GCE and PABSA/AuNP/GCE, it was found that the oxidative catalytic performance of PABSA/AuNP/GCE for Tyr is much better. Because Au nanoparticles have the advantages of a large surface area, good biocompatibility and high activity, the PABSA/AuNP composite film showed better catalytic properties for Tyr than the PABSA film.

Fig. 2(c) shows the CV responses of GA (0.1 mM) on different electrodes. There was no peak current for GA on the GCE and CuNP/GCE surface based on CV measurements, but the response current of CuNP/GCE was much larger than that of the GCE due to the large surface area and good conductivity of Cu nanoparticles. Irreversible oxidation peaks of PGA/GCE and PGA/CuNP/GCE were presented due to the deposition of PGA film. The peak current of PGA/CuNP/GCE was a little larger than that of PGA/GCE, which indicated that PGA/CuNP/GCE showed better catalytic properties for GA.

### 3.3. Optimization of electrochemical parameters

The electrochemical behavior at the surface of the modified electrodes has a high correlation with pH, scan rate and the accumulation step. The electrochemical features of electroactive molecules are dependent on the pH value of the medium. The kinetics of the electrode reaction can be investigated by studying the effects of the scan rate on the redox reaction occurring at the electrode surface. The accumulation step is an effective way to increase the efficiency of detection, especially for adsorption-controlled electrochemical reactions. The optimization of the experimental parameters for PACBK/AuNP/GCE, PABSA/AuNP/GCE and PGA/CuNP/GCE is therefore discussed as follows.

#### 3.3.1. Optimization of pH values

**Fig. 3:**
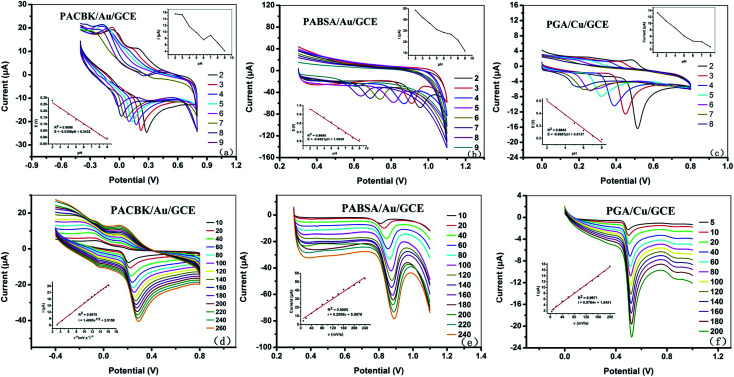
Cyclic voltammograms of (a) Asc on the surface of PACBK/AuNP/GCE, (b) Tyr on the surface of PABSA/AuNP/GCE and (c) GA on the surface of PGA/CuNP/GCE with different pH values. The insets show the relationship of the peak potential and the peak current with the pH values. Electrochemical response curves of PACBK/AuNP/GCE (d), PABSA/AuNP/GCE (e) and PGA/CuNP/GCE (f) at different scan rates. The insets show the relationship of the peak current with the scan rate.

The effect of the pH values on the oxidation peak potential of Asc, Tyr and GA was investigated. It can be seen from Fig. 3(a) that the oxidation peak current of Asc on PACBK/AuNP/GCE decreased gradually with an increase in pH from 2–9, and the oxidation peak potential shifted towards the negative direction significantly. This change in the oxidation peak potential showed that protons are involved in the oxidation of Asc. The general pH of rice wine is from 3 to 5, and the electrode showed better catalysis responses on Asc with a pH of 3. The two insets represent the changes of the peak potential, E, and peak current, I, with the pH. It can be seen that the peak potential, E, had a linear relationship with the pH, with a slope of −33.9, approximate the theoretical slope value (−58.6). In consideration of the experimental error, the oxidation of Asc on the electrode was an isohydric and isoelectronic transformation. The relationship of the change in peak potential and peak current with pH for Tyr was almost the same as that for Asc (Fig. 3(b)). pH 3 was selected for the detection of Tyr throughout the experiments, and the changing tendency of the oxidation peak potential showed that the electrochemical behavior of Tyr was also an isohydric and isoelectronic transformation. Fig. 3(c) shows that the catalytic effect of PGA/CuNP/GCE on GA at different pH values was the same as that of the other two electrodes. With the increase in pH, the peak current decreased gradually and the oxidation peak potential shifted towards the negative direction significantly, with wider peak deformation. pH 3 was also selected for the detection of GA.

#### 3.3.2. Optimization of the scan rates

The influence of the scan rate was investigated in order to clarify the electrochemical processes of Asc, Tyr and GA on PACBK/AuNP/GCE, PABSA/AuNP/GCE and PGA/CuNP/GCE, respectively (Fig. 3(d)–(f)). As shown in Fig. 3(d), the oxidation peak current of Asc on PACBK/AuNP/GCE increased continually with the increase in scan rate, and the peak current was found to be proportional to the square root of the scan rate (Fig. 3(d)). The linear regression equation was *i*_p_ (μA) = 1.4095*v*^1/2^ + 2.0158 (*R*^2^ = 0.9978). Therefore, the redox process of Asc on the PACBK/AuNP/GCE surface was a typical diffusion-controlled electrochemical process. It can be seen from Fig. 3(e) and (f) that both oxidation peak currents from PABSA/AuNP/GCE and PGA/CuNP/GCE linearly scaled with the scan rate, and the linear regression equations were *i*_p_ (μA) = 0.2096*v* + 5.0976 (*R*^2^ = 0.9865) and *i*_p_ (μA) = 0.0764*v* + 1.8451 (*R*^2^ = 0.9971), respectively. The correlation of the peak current with the scan rate indicated that the oxidation processes of Tyr and GA on the electrode surfaces were typical adsorption-controlled electrochemical processes. The charging currents were increased with the increasing scan rate, however, a higher scan rate led to the faster consumption of the modified materials and a lower SNR (signal to noise ratio). Therefore, a median scan rate, 100 mV s^−1^, was applied as the proper scan rate in the experiments.

#### 3.3.3. Optimization of the accumulation potentials and time

**Fig. 4:**
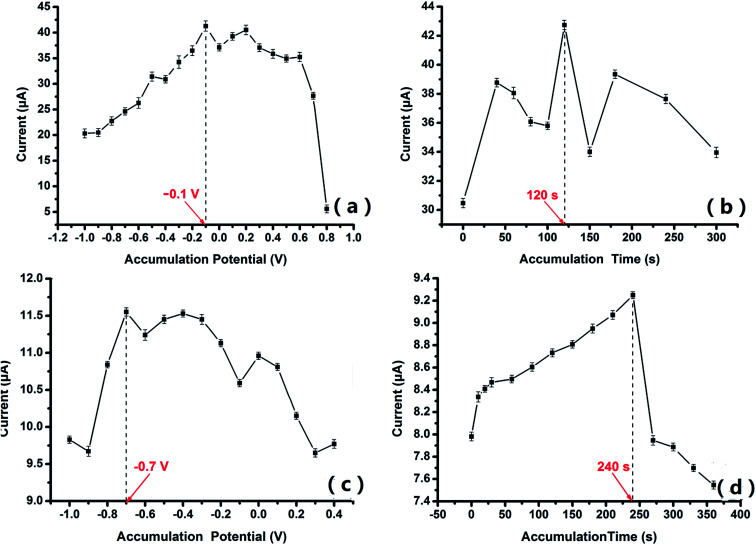
The change in peak current with the accumulation potential and time during the experiments for Tyr (a and b) and GA (c and d).

The oxidation of Tyr and GA on PABSA/AuNP/GCE and PGA/CuNP/GCE are typical adsorption-controlled processes which are influenced by the accumulation potentials and time. Fig. 4(a) and (b) show the tendency of the peak currents of Tyr to change with the accumulation potential and time, recorded using CV. As shown in Fig. 4(a), the peak currents of Tyr increased with an increase in the accumulation potential from −1.0 V to −0.1 V, and they remained stable in the range of −0.1 V to 0.1 V. From 0.1 V to 0.8 V, the peak currents decreased with the increase in accumulation potential. Therefore, −0.1 V was selected as the best accumulation potential. It can be seen from Fig. 4(b) that the peak current of Tyr was stable when the accumulation time was over 60 s, and it reached its maximum value at the accumulation time of 120 s. 120 s was therefore selected as the best accumulation time. Fig. 4(c) and (d) show the tendency of the peak currents of GA to change with the accumulation potential and time. The peak current reached its maximum value when the accumulation potential was −0.7 V. −0.7 V was therefore applied as the working accumulation potential in the experiment (Fig. 4(c)). The peak currents increased with time for 240 s (Fig. 4(d)), but showed a sharp downward trend after 240 s, which might be the reason that electrodes with long periods of accumulation generate their own losses and thus decrease in stability. 240 s was therefore selected as the best accumulation time.

### 3.4. Quantitative detection of chemical substances

**Fig. 5:**
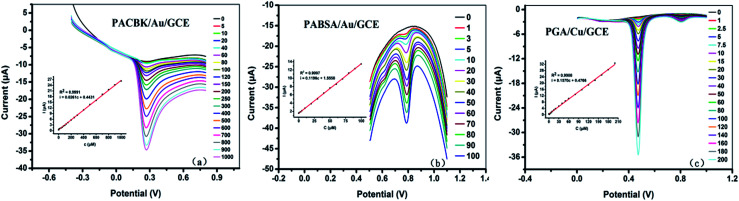
The quantitative detection of Asc, Tyr and GA on (a) PACBK/AuNP/GCE, (b) PABSA/AuNP/GCE and (c) PGA/CuNP/GCE, respectively. The inset shows the calibration curve of the peak currents and concentration.

As shown in Fig. 5(a), the Asc solution (PBS solution, pH = 3) from 5 × 10^−6^ to 1 × 10^−3^ M was detected quantitatively with differential pulse voltammetry (DPV) based on these optimal conditions. The corresponding oxidation peak current increased with the increase of Asc concentration. The inset illustrates a good linear relationship between the peak current and concentration. The linear regression equation was *i*_p_ (μA) = 0.0261*c* (μM) + 0.4431 (*R*^2^ = 0.9991), and the limit of detection (LOD) was calculated to be 0.35 mg L^−1^ based on a signal-to-noise ratio of 3 (S/N = 3).

The Tyr solution from 1 × 10^−6^ to 1 × 10^−4^ M was detected quantitatively by DPV at a potential range of 0.5 V to 1.1 V (Fig. 5(b)). The peak current also increased with the increasing Tyr concentration. The inset shows a good linear relationship between the peak current and concentration. The linear regression equation was *i*_p_ (μA) = 0.1196*c* (μM) + 1.5558 (*R*^2^ = 0.9997), and the LOD was calculated to be 0.07 mg L^−1^ (S/N = 3).

The GA solution from 1 × 10^−6^ to 2 × 10^−4^ M was detected quantitatively by DPV at a potential range of 0 to 1.0 V (Fig. 5(c)). The peak current increased with the increase of solution concentration, and the inset shows a good linear relationship between the peak current and concentration. The linear regression equation was *i*_p_ (μA) = 0.1570*c* (μM) + 0.4766 (*R*^2^ = 0.9994), and the LOD was calculated to be 0.05 mg L^−1^ (S/N = 3). According to the linear equations above, the concentration of the chemical substance solution could be calculated. It therefore provided a theoretical basis for the three types of modified electrode to be applied to the analysis of rice wine samples.

### 3.5. Electrochemical features of the modified electrodes for rice wine of different brands

#### 3.5.1. Response curves of the modified electrodes for rice wines

It was obvious that PACBK/AuNP/GCE, PABSA/AuNP/GCE and PGA/CuNP/GCE showed high sensitivity to Asc, Tyr and GA, which have high correlations with wine brands. As shown in Table 2, the concentrations of the three chemicals in rice wines were higher than the limits of detection (LOD). The three chemicals may not be detected quantitatively due to matrix effects, however, the electrochemical behavior of Asc, Tyr and GA on the surface of the modified electrodes and the electronic information of Asc, Tyr and GA could be obtained using the electrodes. The information obtained by the three modified electrodes for rice wines was more abundant than that obtained by the bare GCE, and it included information for Asc, Tyr and GA, which have good correlation with rice wine brands. Therefore, the potential of polymer/metal nanocomposite-modified electrodes for the identification of rice wines of different brands was evaluated in this work.

**Fig. 6:**
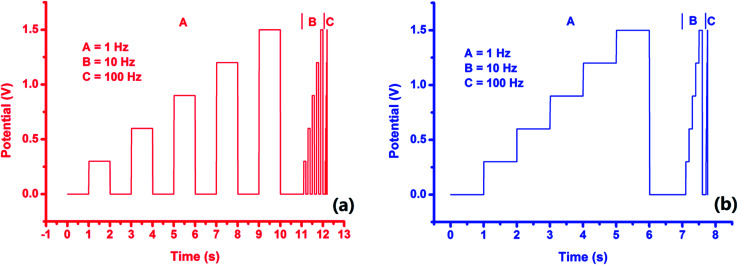
The applied multi-frequency and potential step scanning waveforms for rectangle voltammetry (a) and staircase voltammetry (b).

In this paper, the chronoamperometry method included MRPV and MSPV, which were composed of three frequencies of 1 Hz, 10 Hz and 100 Hz, and they were applied as the scanning potential waveforms (Fig. 6). Fig. S1† shows the response current curves obtained by PACBK/AuNP/GCE, PABSA/AuNP/GCE and PGA/CuNP/GCE from rice wines of 7 brands. Fig. S1(a) and (b)† shows the response current curves obtained using PACBK/AuNP/GCE. The response curves could not be distinguished from each other because the rice wines were all produced in Shaoxing with the same marked age, and the Asc content was lower in these rice wines. Fig. S1(c) and (d)† show the response current curves obtained by PABSA/AuNP/GC. The response curves of the rice wines could be distinguished from each other. The response currents from large to small were G-DD, G-GF, T-SG, G-KC, T-LJ, T-TC and K-HD. The corresponding response currents of rice wine at the tenth second were −307.9, −147.4, −90.8, −78.3, −62.2, −44.1 and −31.9 μA (Fig. S1(c)†), respectively. The corresponding response currents at the sixth second were −191.0, −102.6, −65.8, −57.1, −45.6, −32.2 and −24.4 μA (Fig. S1(d)†), respectively.

Fig. S1(e) and (f)† show the response current curves obtained by PGA/CuNP/GCE based on the optimal conditions. All of the curves could not be distinguished obviously from each other, except for the larger response current of G-DD. The information obtained by each modified electrode with a single scanning waveform provided the complete brand information for the rice wines, and this information was applied for classification and prediction based on pattern recognition methods.

#### 3.5.2. Stability evaluation and variance analysis of the modified electrodes

The variable coefficients of the area feature data obtained by the three modified electrodes are shown in the Table 3. The mean values and standard deviation values were calculated using the response values obtained from the modified electrodes based on MRPV and MSPV from 40 samples of the same brand. 7 types of variable coefficient were used to evaluate the stability of each electrode. It was observed that all of the variable coefficients were less than 7%, and the stability of all electrodes met the requirements in the experiments.

To evaluate the effect of the waveforms and modified electrodes on the response values, respectively, two-factor analysis of variance of the scanning waveforms (rectangle wave and staircase wave) and modified electrodes (PACBK/AuNP/GCE, PABSA/AuNP/GCE and PGA/CuNP/GCE) was performed based on the area feature data. The results of the analysis of samples of Kuaijishan are shown in Table 4. Smaller *P* values (*P* < 0.05) and larger *F* values indicated a more significant effect on the corresponding response variables. The *F* values of the scanning waveform and modified electrode were 14 757.624 and 18 174.932, respectively, and 3478.538 when the scanning waveform and modified electrode interacted. All of the *P* values were below 0.05. ANOVA analysis showed that the waveforms and electrodes, and the interaction of both, had a significant effect on the response, and that a detection system composed of two waveforms and three modified electrodes could be applied to analyze rice wine samples.

### 3.6. The classification and prediction of rice wines of different brands

The area surrounded by the response curve and the abscissa axis was applied as feature data which was used for the classification and prediction of rice wine brands using pattern recognition methods. The processing procedures were as follows. Firstly, the correlation of the feature data with the wine brand was determined using the area method from the original responses. Secondly, the feature data were reduced to two dimensions by different dimensional reduction methods, and the efficiency of the dimensional reduction methods was compared using classification plots. Finally, the data compressed by the most efficient dimensional reduction method were applied as the input data in the prediction models for wine brand prediction. In this paper, PCA (an unsupervised method),^44^ LPP (a semi-supervised method)^45^ and LDA (a supervised method)^46^ were applied to reduce the dimensions of the original feature data, and these methods also displayed the classification results of the different brands of rice wine in two-dimensional or three-dimensional graphs. ELM,^47^ LIBSVM^48^ and BPNN^49^ were applied to predict the rice wine brands. The best method for this experiment was selected according to the effect of various models.

#### 3.6.1. Results of the classification of rice wines

Fig. S2(a)† shows the results of PCA for the discrimination of rice wines of different brands. The first two dimensions of the principal component explained the variance data of 73.24% and 21.25%, respectively, whose accumulative contribution rate was 94.49%. Seven types of rice wine were separated clearly, except for Kuaijishan and Tapai 2. Three types of Tapai and Kuaijishan sample had a centralized distribution, but the Guyue Longshan samples were dispersed. Moreover, all of the samples could be separated into two clusters: (I) the Tapai and Kuaijishan samples which were gathered on the left side of the PC1 axis, and (II) the Guyue Longshan samples which were gathered on the right side of the PC1 axis. The samples, which contain similar chemical substances, could be grouped together in the score plot. The regular distribution of the samples confirmed the potential of the modified electrode array for the identification of rice wines. The cumulative discrimination contribution rates of the first two dimensions of LPP and LDA were 97.75% and 99.56% (Fig. S2(b) and (c)†), respectively. LPP and LDA presented the same distribution of samples as PCA. A weakness of these classification methods was that misjudgment might occur because the rice wines of Tapai and Kuaijishan could not be separated clearly.

#### 3.6.2. Results of the prediction of rice wine brands

ELM, LIBSVM and BPNN were applied for the prediction of wine brands. As shown in Table 1, each type of sample was given a reference value which was applied as the dependent variable for predictive analysis. Overall, 280 samples (40 samples of each brand) in the experimental session were divided randomly into calibrating and test subsets: 175 samples (25 samples of each brand) for the training set and 105 samples (15 samples of each brand) for the testing set.

**Table 1:** The experimental sample selection scheme of different brands of rice wines

No.	Brand	Marked age (years)	Number of samples	Reference value for prediction
1	Guyue Longshan Dida (G-DD)	5	40	1
2	Guyue Longshan Gold Five (G-GF)	5	40	2
3	Guyue Longshan Kucang (GKC)	5	40	3
4	Tapai Techun (T-TC)	5	40	4
5	Tapai Laojun (T-LJ)	5	40	5
6	Tapai Shougong (T-SG)	5	40	6
7	Kuaijishan Huadiao (K-HD)	5	40	7

**Table 2:** Comparison of the LOD of the electrodes with the corresponding substance content in the rice wines

Electrode	Detected substance	LOD (mg L^−1^)	Content (mg L^−1^)
PACBK/AuNP/GCE	Asc	0.35	5.71–43.20
PABSA/AuNP/GCE	Tyr	0.07	70–1979.45
PGA/CuNP/GCE	Glc	0.05	8246–43 195

**Table 3:** Evaluation of the stability (variable coefficient) of the modified electrodes

Electrode	Rice wine	Rectangle wave			Staircase wave			Average variable coefficient
		Mean	Standard deviation	Variable coefficient	Mean	Standard deviation	Variable coefficient	
PACBK/AuNP/GCE	G-DD	0.0888	0.0045	5.03%	0.0356	0.0022	6.28%	4.64%
	G-GF	0.0756	0.0032	4.27%	0.0310	0.0017	5.53%	
	G-KC	0.1100	0.0064	5.84%	0.0426	0.0027	6.28%	
	T-TC	0.0662	0.0022	3.30%	0.0272	0.0012	4.59%	
	T-LJ	0.0624	0.0019	3.05%	0.0258	0.0011	4.37%	
	T-SG	0.0705	0.0023	3.32%	0.0290	0.0014	4.95%	
	K-HD	0.0599	0.0021	3.59%	0.0249	0.0011	4.55%	
PABSA/AuNP/GCE	G-DD	1.3599	0.1032	7.59%	0.4778	0.0389	8.13%	6.85%
	G-GF	0.8039	0.0329	4.10%	0.2966	0.0229	7.74%	
	G-KC	0.5082	0.0543	10.69%	0.1873	0.0246	13.11%	
	T-TC	0.4091	0.0148	3.62%	0.1552	0.0118	7.63%	
	T-LJ	0.2918	0.0111	3.81%	0.1122	0.0086	7.66%	
	T-SG	0.5693	0.0202	3.55%	0.2134	0.0162	7.58%	
	K-HD	0.2153	0.0077	3.56%	0.0857	0.0061	7.10%	
PGA/CuNP/GCE	G-DD	0.1261	0.0202	15.99%	0.0434	0.0031	7.15%	6.12%
	G-GF	0.0756	0.0032	4.25%	0.0303	0.0015	5.03%	
	G-KC	0.0691	0.0113	16.36%	0.0282	0.0043	15.25%	
	T-TC	0.0610	0.0010	1.59%	0.0252	0.0010	3.78%	
	T-LJ	0.0579	0.0009	1.54%	0.0239	0.0009	3.62%	
	T-SG	0.0658	0.0013	2.00%	0.0271	0.0011	4.13%	
	K-HD	0.0560	0.0008	1.39%	0.0233	0.0008	3.57%	

**Table 4:** Two-factor analysis of variance based on area feature data

Variation source	Quadratic sum	Degree of freedom	Mean square value	*F*	*P*
Calibration model	1.021	5	0.204	11 612.913	0.000
Intercept	1.442	1	1.442	81 948.320	0.000
Waveform	0.260	1	0.260	14 757.624	0.000
Electrode	0.639	2	0.320	18 174.932	0.000
Waveform^a^ electrode	0.122	2	0.061	3478.538	0.000
Error	0.004	234	0.000		
Total variation	2.467	240			
Correction variation sum	1.026	239			

a *P* < 0.05 level, *R*^2^ = 0.996, correction *R*^2^ = 0.996.

The results of prediction using ELM, LIBSVM and BPNN were evaluated by the correlation coefficient (*R*^2^) and root mean square error (RMSE) (a good result should have a low RMSE, close to zero, and a high correlation coefficient, close to one). The radial basis function (RBF) was chosen as the kernel function of ELM for prediction, and the number of neurons of the hidden layer was set from 5 to 100. As shown in Fig. S3(a),† the neuron number of the hidden layer was 45 when the prediction model presented the best results, and the results of wine brand prediction based on the training and testing datasets were *R*^2^ = 0.9665, RMSE = 0.3720 and *R*^2^ = 0.9620, RMSE = 0.4060, respectively (Table 5). Although both *R*^2^ values met the requirements, both RMSE values were not good enough. RBF was also applied as the kernel function of LIBSVM, and the important parameter *g* and the penalty factor *c* were auto calculated (*g* = 1 and *c* = 32, Fig. S3(b)†). As shown in Table 2, the results of wine brand prediction based on the training and testing datasets were *R*^2^ = 0.9762, RMSE = 0.3134 and *R*^2^ = 0.9621, RMSE = 0.4147, respectively. The prediction result was similar to that of ELM, whose RMSE was not good enough. Although all of the correlation coefficients (*R*^2^) of ELM and LIBSVM based on the training and testing datasets were over 0.96, the values of the RMSE were large, and all of the RMSE values of the testing dataset were 0.40. According to the analyses above, both prediction models were not the best choice. For a better result, BPNN, with three layers, was applied to predict wine brands. The network topology of BPNN was 6–30–7, *i.e.* the node number in the input layer was 6 (6 types of feature data), in the hidden layer was 30 and in the output layer was 7. The results using BPNN based on the training dataset and testing dataset were *R*^2^ = 0.9952, RMSE = 0.1378 and *R*^2^ = 0.9827, RMSE = 0.2673, respectively (Table 2). It was obvious that the results of prediction using BPNN showed the highest *R*^2^ values and the lowest RMSE values, which indicated that BPNN was the best method to predict wine brands, better than ELM and LIBSVM.

**Table 5:** The results of prediction using ELM, LIBSVM and BPNN

	ELM		LIBSVM		BPNN	
	*R* ^2^	RMSE	*R* ^2^	RMSE	*R* ^2^	RMSE
Training set	0.9665	0.3720	0.9762	0.3134	0.9952	0.1378
Testing set	0.9620	0.4060	0.9621	0.4177	0.9827	0.2673

## 4. Conclusions

Three types of polymer/metal nanocomposite-modified electrode were self-fabricated for classifying and predicting wines of different brands. SEM images exhibited clearly that the synthetic process of the composite could inhibit the aggregation of the metallic nanoparticles, and the electrochemical parameters of the three modified electrodes (pH, scan rate, accumulation potential and time) were optimized using CV and LSV. The modified electrodes showed stable performance for detecting rice wines based on MRPV and MSPV. With the help of pattern recognition methods, all of the samples could be separated using PCA, LPP and LDA, and the samples containing similar chemicals could be grouped together in the score plots. BPNN performed better than ELM and LIBSVM for the prediction of wine brands, and the prediction results were *R*^2^ = 0.9952, RMSE = 0.1378 (training set) and *R*^2^ = 0.9827, RMSE = 0.2673 (testing set). The results of the study show that the polymer/metal nanocomposite-modified electrode is a promising and practical tool for the classification of rice wines. In the future, more effort should be put into fabricating more sensitive electrodes and optimizing the signal processing algorithm.

## Conflicts of interest

There are no conflicts to declare.

## Supplementary Material

RA-008-C8RA00164B-s001
