# Supplementary material for: Application of novel nanocomposite-modified electrodes for identifying rice wines of different brands
Source: RSC Adv. 2018 Apr 10;8(24):13333–43. doi: 10.1039/c8ra00164b (PMC9079784; doi:10.1039/c8ra00164b)
Supplement: RA-008-C8RA00164B-s001 [file RA-008-C8RA00164B-s001.pdf]

Electronic Supplementary Material (ESI) for RSC Advances.
This journal is © The Royal Society of Chemistry 2018

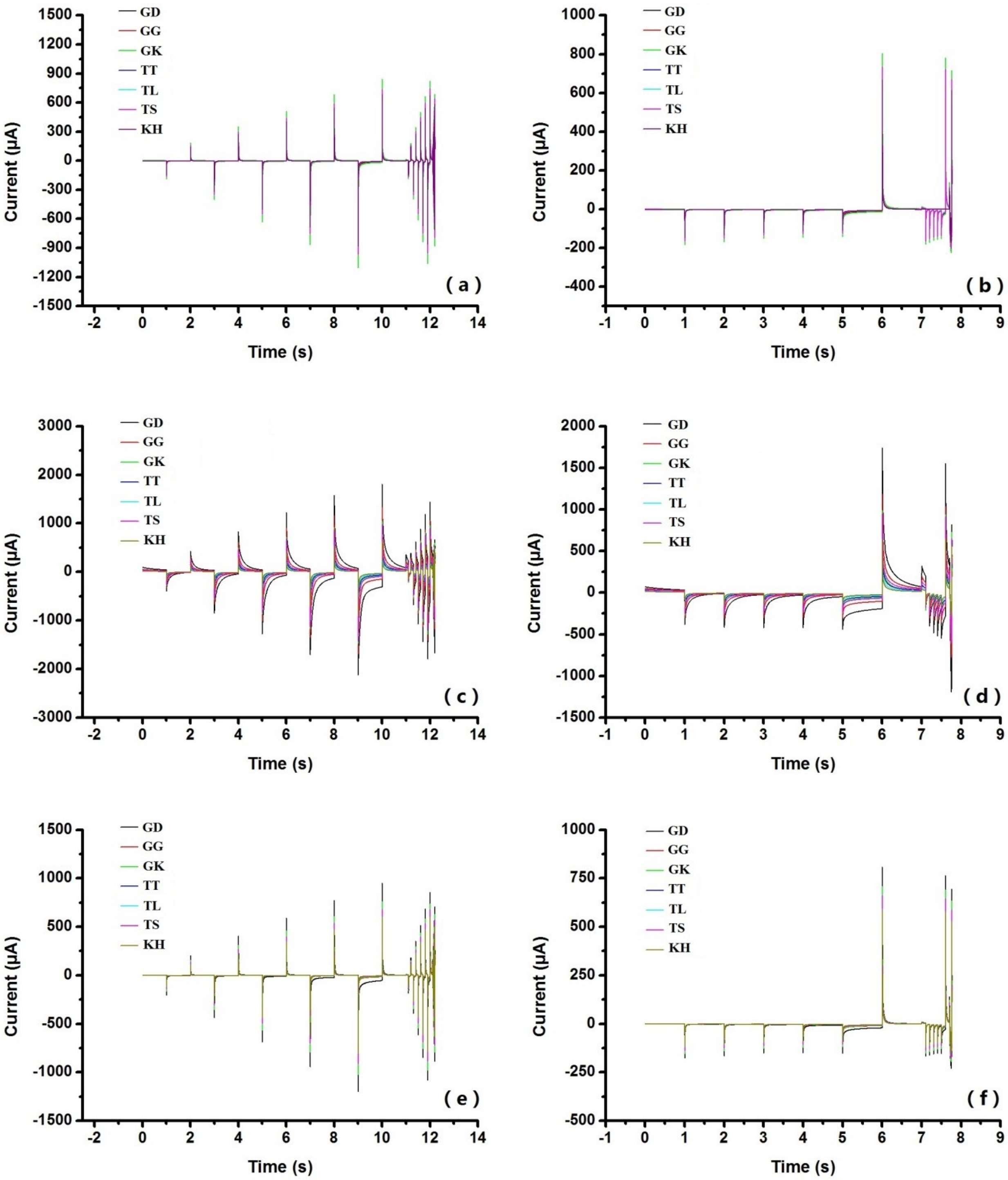

Fig. S1 The response current curves of modified electrodes array to 7 brands of rice wines. The responses obtained by PACBK/AuNPs/GCE based on the MRPV (a) and MSPV (b), the responses obtained by PABSA/AuNPs/GCE based on MSPV (c) and MSPV (d), the responses obtained by PGA/CuNPs/GCE based on MSPV (e) and MSPV (f).

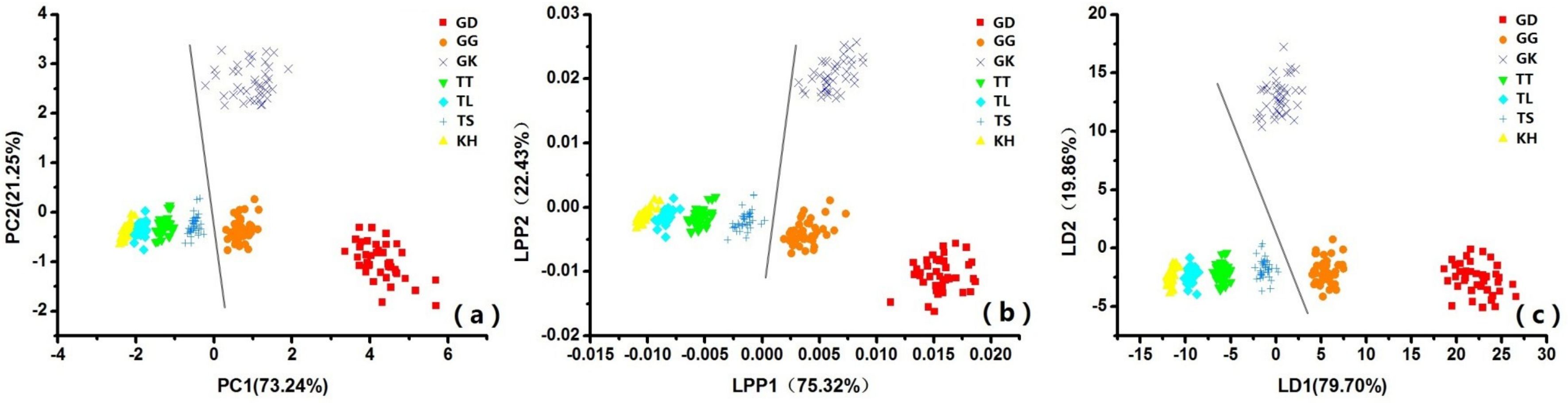

Fig. S2 Discrimination results of (a) PCA, (b) LPP and (c) LDA based on area feature data.

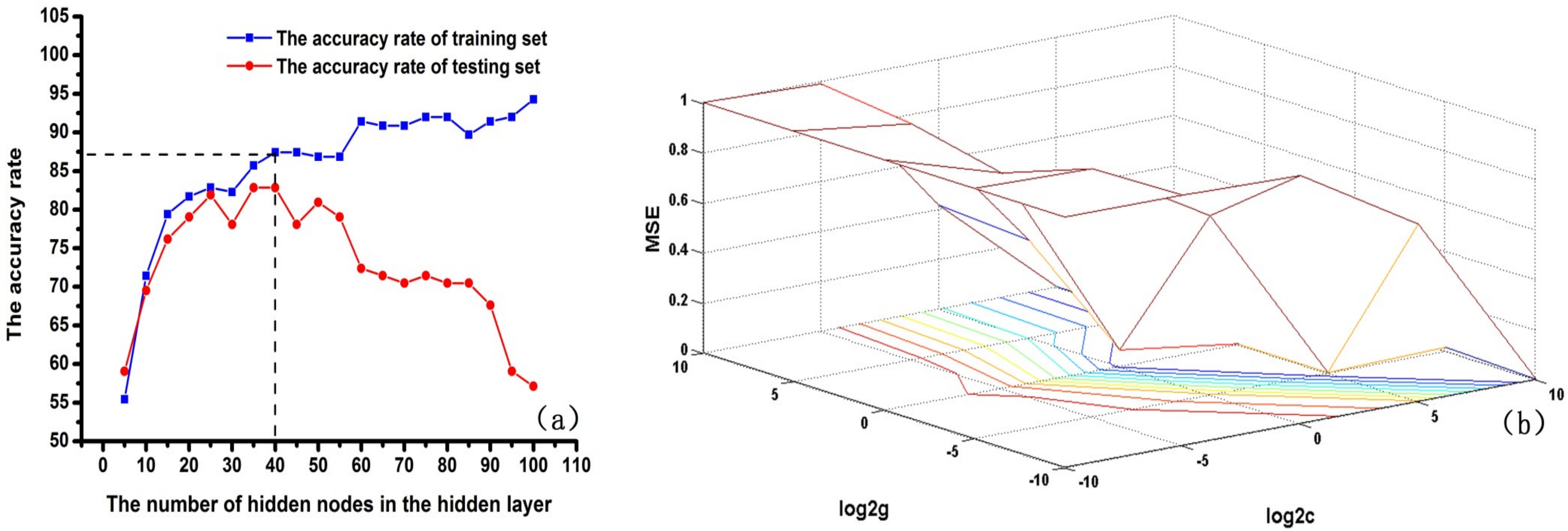

Fig. S3 The variation trend of the accuracy rate of ELM model (a) and the 3D view of parameter selections of LIBSVM (b).
